# Dataset on biodiversity and agronomic performance of lentil and chickpea field trials in the Mediterranean Region

**DOI:** 10.1016/j.dib.2024.110528

**Published:** 2024-05-15

**Authors:** Anna-Lena Vollheyde, Dulcenombre Rodriguez Navarro, Marcelino de los Mozos Pascual, María Cristina Alcántara Ramírez, Sanja Sikora, Ivana Rajnović, Imran Hammami, Darine Trabelsi, Christina von Haaren

**Affiliations:** a Institute of Environmental Planning, Leibniz University Hannover, Herrenhäuser Straße 2, 30419 Hannover, Germany; b IFAPA- Centro Las Torres, Ctra. Sevilla-Cazalla, Km 12.2 41200-Alcalá del Río, Seville, Spain; c Regional Institute of Agri-Food and Forestry Research and Development of Castilla-La Mancha (IRIAF). CIAF Albaladejito. Ctra. Toledo-Cuenca (CN 400), km 174. 16194 Cuenca. Spain; d University of Zagreb Faculty of Agriculture, Svetošimunska cesta 25, 10000 Zagreb, Croatia; e LR: Bioressources, Environment and Biotechnology – ISSBAT. Tunis El Manar University, Tunisia; f LR: Legumes and Sustainable Agrosystems, Biotechnology Center, Borj-Cédria Technopole (CBBC) - BP 901, 2050. Hammam-lif, Tunisia

**Keywords:** Flora, Biodiversity, Pulses, Intercropping, Inoculation

## Abstract

Pulse crops have become more important in food production and consumption systems for the transition towards sustainability. We present an agroecological dataset from 304 samples from 12 legume field trials in five locations across three countries in the Mediterranean. The field trials were established in the seasons 2021/22 and 2022/23 and tested different lentil or chickpea cultivars, inoculants, intercropping and weeding regimes. The dataset encompasses detailed information on wild flora diversity, grain yield, associated management practices, soil texture and weather during the growing period. Wild flora diversity was recorded by conducting a vegetation survey in 1 × 2 m sample plots. Grain yield was determined at the crop maturity stage, with full plots harvested in Spain, while samples were taken in Croatia and Tunisia. Environmental variables were via laboratory analysis or bottle testing of soil samples and analysis of local weather data. The comprehensiveness of the dataset, including all relevant agroecological information, enables other researchers to employ the dataset for various statistical analyses of agroecosystem processes, such as plant-environment interactions or biodiversity-yield trade-off analysis.

Specifications Table

**Table:** 

Subject	Biodiversity, Agronomy and Crop Science
Specific subject area	Flora biodiversity and agronomic performance of lentil and chickpea field experiments
Data format	Raw
Type of data	.xlsx file (data tables and metadata) .pdf files (experimental layouts)
Data collection	Different field trials utilizing lentil and chickpea were established in five regions of three Mediterranean countries in 2021/22 and 2022/23. The tested treatments include legume cultivars, inoculants, intercropping and weeding regimes. In the experimental plots, vegetation surveys were done in summer 2022 and 2023 in 1 × 2 m sample-plots following the drilled lines. Grain yield at the maturity stage was determined in the entire plots in Spain, and via subsamples in Croatia and Tunisia. Apart from the tested treatment, all other management details were synthesized. Soil texture was deduced through laboratory analysis or bottle test. Weather data were retrieved via local weather stations.
Data source location	Carmona, Spain Cuenca, Spain Zagreb, Croatia Saida, Tunisia Korba, Tunisia
Data accessibility	Repository name: Forschungsdaten-Repositorium der Leibniz Universität Hannover Data identification number: https://doi.org/10.25835/lzb211it Direct URL to data: https://data.uni-hannover.de/dataset/flora-diversity-yield-and-agri-environmental-information-legume-field-trials-mediterranean

## 1 Value of the Data

• The presented agroecological dataset comprises wild flora diversity, grain yield, associated management practices, soil, and weather data from different field trials involving lentil or chickpea in different Mediterranean regions.
• The field trials were adapted to local conditions and sought to assess various management approaches to minimize reliance on external inputs, mitigate environmental effects, and prevent diversity loss at genetic and species levels. The treatments included different legume cultivars, inoculants, intercropping and weeding regimes.
• Besides provisioning services, the dataset also presents a systematic recording of associated wild flora diversity in legume systems and its potential changes pertaining to management practices. The documentation of all agricultural management activities, not only the tested practices, alongside with environmental characteristics of the experimental sites, enables other researchers to employ the dataset for a variety of statistical analyses of agroecosystem processes. This includes investigations of relationships such as plant-environment, biodiversity-management, or yield-biodiversity trade-off analysis leading to identifying strategic points for promoting agro-biodiversity conservation and remuneration.

## 2 Background

Against the backdrop of climate change and biodiversity loss, a transition of land-use systems towards more sustainability is greatly needed. For the food system, pulse crops become increasingly important due to, inter alia, their nitrogen fixation ability, nutritional value for human consumption and diversification potential for agroecosystems [[1], [2], [3], [4]]. The systematic review of [5] on the ecosystem service potential of legumes in Europe shows that mostly provisioning services of few legumes are examined in field trials. Less than 1% of the studies address biodiversity and certain pulse crops, including lentil and chickpea, are underrepresented in field studies [5]. Moreover, only few studies examined the impact of wild flora diversity on productive services in legumes systems, whereby they come to differing results [5,6], highlighting further need in research [7]. And also, knowledge on the relation between planned and unplanned diversity in legumes in scarce [8]. Therefore, studying the diversity of wild flora in legume systems and their relationship to changes in management practices together with yields, can help derive leverage points for the management of agroecosystems and the conservation and enhancement of biodiversity in the fields.

## 3 Data Description

**Fig. 1:**
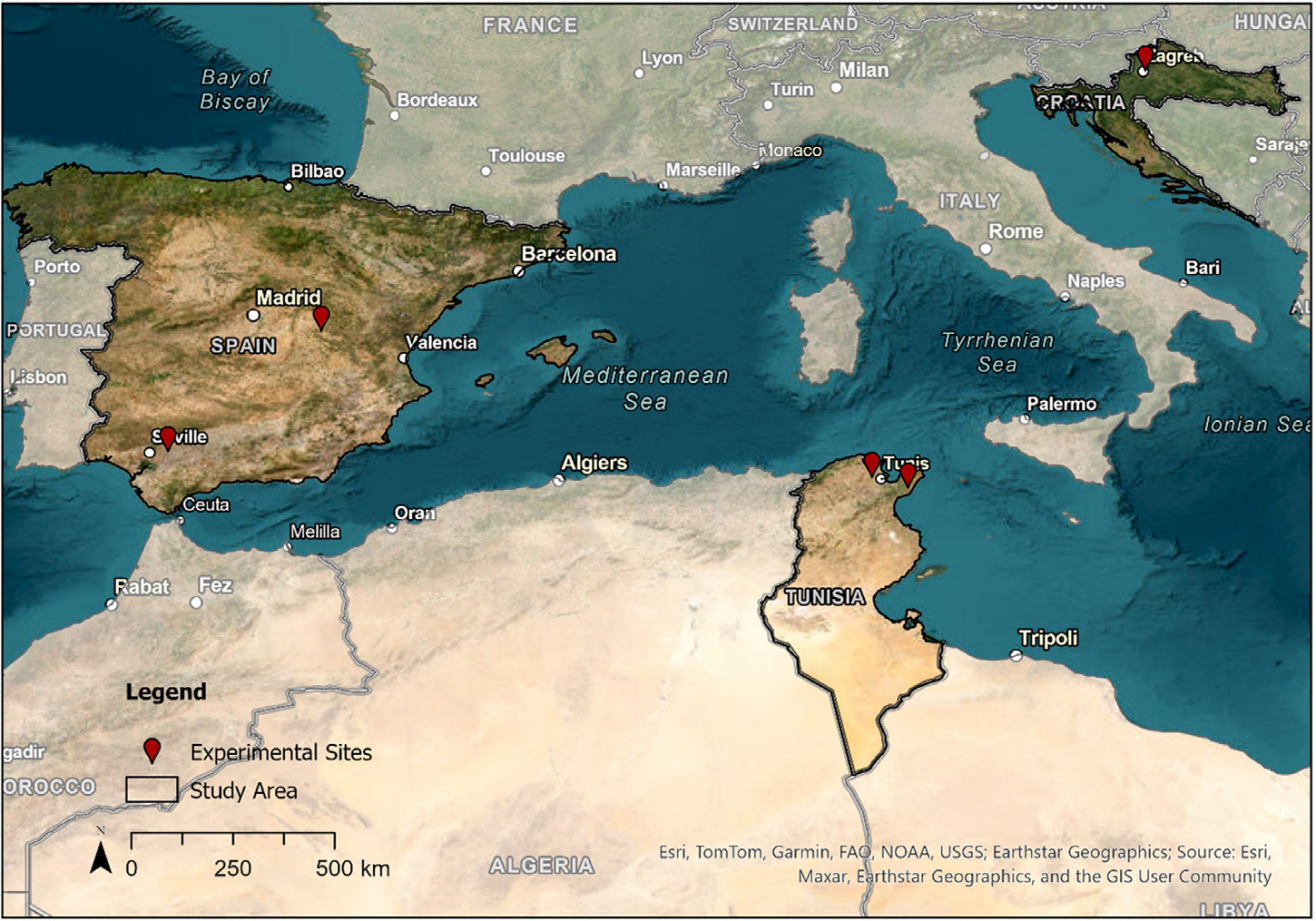
Allocation of the experimental stations.

Here, we describe an agroecological dataset within different contrary management systems tested on experimental sites across the Mediterranean region in 2021/22 and 2022/23 [9]. We recorded wild flora diversity, grain yield, associated management practices, soil, and weather data of different field trials involving lentil or chickpea. The dataset contains 304 samples from 12 field trials in five locations across three countries, which are shown in Fig. 1. The locations encompass two experimental sites in the north of Tunisia (regions Manouba and Nabeul), one site in the more continental part of Croatia (Zagreb) and two sites in Spain (regions Andalusia and Castilla-La Mancha) .

**Fig. 2:**
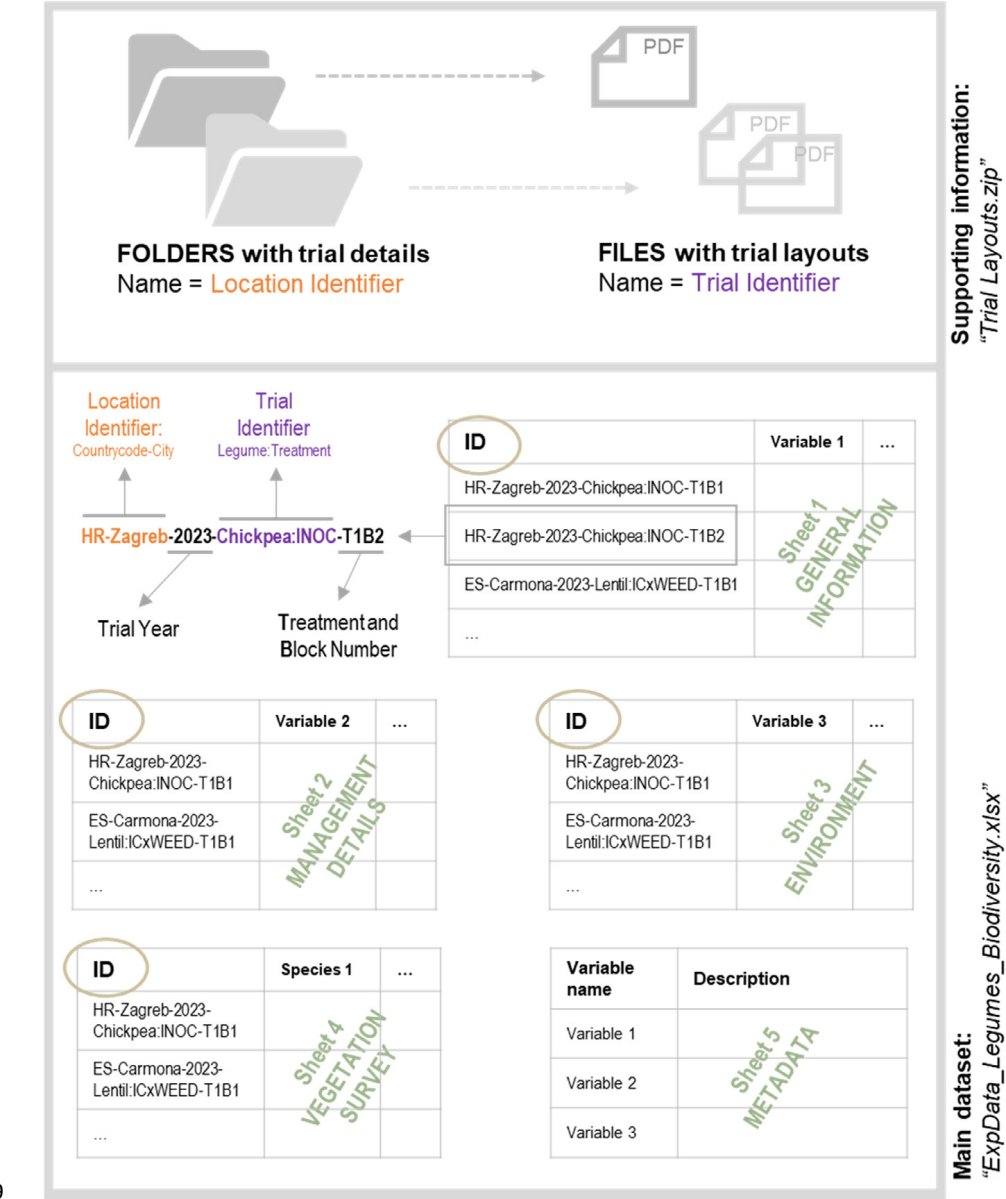
Composition of the dataset and structure on how to join and read the dataset in combination with additional trail information.

The data are organized following a common dataset structure consisting of a main dataset and supplementary information. The structure and interconnection of files are illustrated in Fig. 2. The main dataset is the sampled data being stored in an Excel workbook (“ExpData_Legumes_Biodiversity.xlsx”). It is accompanied by supplementary information about the trial layouts (PDFs) in a ZIP folder.

The workbook containing the sampled data is, for easier readability, split into five sheets based on a grouping of the topic of the variables, as presented in Table 1. The sampled data encompass general information about the field trial (sheet 1), management details including grain yield (sheet 2), environmental data (soil and weather) (sheet 3), and the vegetation survey with the wild flora lists (sheet 4). The 5th sheet summarizes the dataset's metadata by describing the given variables.

**Table 1:** Overview of variables surveyed and the sheet structure in the workbook of the main dataset.

Sheet Name	Variable Abbreviation / Metadata	Description
General Information	Year	Year during which the vegetation survey was carried out
	Country	ISO 3166 Alpha-2 code of the country where the experimental station is located
	Region	Region where the experimental station is located
	Size	Plot size of the experiment/treatment (in m x m)
	DAW	Days after weeding: number of days between the last weeding and the sampling day
	BBCHMax	Maximum BBCH Growing stage of the legumes within the sample-plot at the sampling day

Management Details	SowD	Sowing date
	HarD	Harvest date
	Leg	Grown legume crop
	CropIC	Other crop grown in intercropping trials
	PrevCrop	previous crop grown on the site before the trial
	CT	Cropping type (intercropping, monocropping)
	Till	Tillage type (conventional, conservation or no tillage)
	TillD	Tillage depth or soil working depth (in cm)
	Betw	Sowing distance between rows (in cm)
	With	Sowing distance within rows (in cm)
	Depth	Sowing depth (in cm)
	WeedApl	Total number of weeding operations during the legume phase (= from seedbed preparation to harvest)
	Weed	Weeding type (none, mechanical, hand weeding)
	Inoculation	Active inoculation of seeds or soil with rhizobia (yes, no)
	Pass	Number of passings with machinery on the field to conduct all needed operations for legume cultivation during the legume phase (= from seedbed preparation to harvest); This variable is a proxy for the management intensity.
	LegYield	Legume grain yield (in kg/ha)
	TotYield	Total grain yield (in kg/ha)
	LegCov	Estimated legume cover in % within the sample-plot
	CropICCov	Estimated legume cover of the other crop grown in intercropping trials in % within the sample-plot
	TotalCov	Total plant cover (crops and wild flora) within the sample plot (in %)

Environment	Sand	Total sand content (in %)
	Clay	Total clay content (in %)
	Silt	Total silt content (in %)
	Temp	Average temperature during legume growing phase (= from sowing to harvest date) (in°C)
	Precip	Total sum of rainfall during legume growing phase (= from sowing to harvest date) (in mm)

Vegetation Survey	Species Name	Name of the species and its estimated cover (in %) within the sample-plot
	SpeciesRichness	Total species richness of wild flora within the sample-plot
	FloraCov	Total estimated wild flora cover in % within the sample-plot

Metadata Variable Description	Further details on each variable is presented here.	

All sheets in the workbook, except for the metadata table, can be connected through the “ID” field. The ID field is a distinct identifier corresponding to information gathered from a single sample. The ID is composed of several sub-identifiers: the location identifier Countrycode-City (e.g. HR-Zagreb), the trial year (e.g. 2023), the trial identifier Legume:TREATMENT (e.g. Chickpea:INOC) and the treatment and block number (e.g. T1B1 for parcel of treatment 1 in block 1). The sub-identifiers help correctly connect the relevant information from the supplementary information (trial layouts) with the respective database entries in the workbook. Fig. 2 illustrates this principle. The supplementary ZIP folder with trial layouts is organised in sub-folders named after the location identifier. Within the folders, a separate PDF containing the trial layout details can be found for each experimental trial of each region. The PDFs are named after the respective trial identifiers. Thus, with the folder and file name of the supplementary information the trial layouts can be correctly matched to the corresponding database entries in the excel workbook.

## 4 Experimental Design, Materials and Methods

### 4.1 Field trials

Field experiments utilizing lentil or chickpea were carried out aiming to test different management practices to reduce the use of external inputs and limit environmental impacts and as well as the loss of crop-diversity (genetic and species level) under different climatic conditions. Within each country, different field trials have been tested, considering local agronomic and climatic circumstances. An overview of the tested treatments can be found in Table 2, and the experimental layouts are provided in the dataset. All in all, the trials tested the performance of different legume cultivars, different legume-rhizobia combinations, as well as intercropping. Additionally, plots without weeding have been set up in different locations to allow for estimating the site's basic wild flora biodiversity potential in the legume system.

**Table 2:** Overview of the different treatments tested within the field experiments.

Country	Season	City	Legume		Treatments tested
			Chickpea	Lentil	
Spain	2021/22	Carmona	x		Legume cultivar x pre-crop (x weeding)
				x	Legume cultivar x intercropping x inoculation
	2022/23	Carmona		x	Legume cultivar x intercropping x inoculation
				x	Intercropping x weeding
		Cuenca		x	Legume cultivar x intercropping x inoculation
				x	Intercropping x weeding

Tunisia	2021/22	Saida	x		Cultivar x P fertilization x weeding
	2022/23	Korba	x		Weeding

Croatia	2021/22	Zagreb		x	Inoculation x intercropping
			x		Inoculation x intercropping
	2022/23	Zagreb	x		Inoculation (and no weeding)

### 4.2 Data sampling

The data sampling encompassed on-field vegetation survey, grain yield recording, and post-hoc data acquisition through management synthesis and acquisition of weather and soil data. The choice of variables to be studied was based on theoretical and empirical findings from literature such as [[10], [11], [12], [13], [14]].

**Fig. 3:**
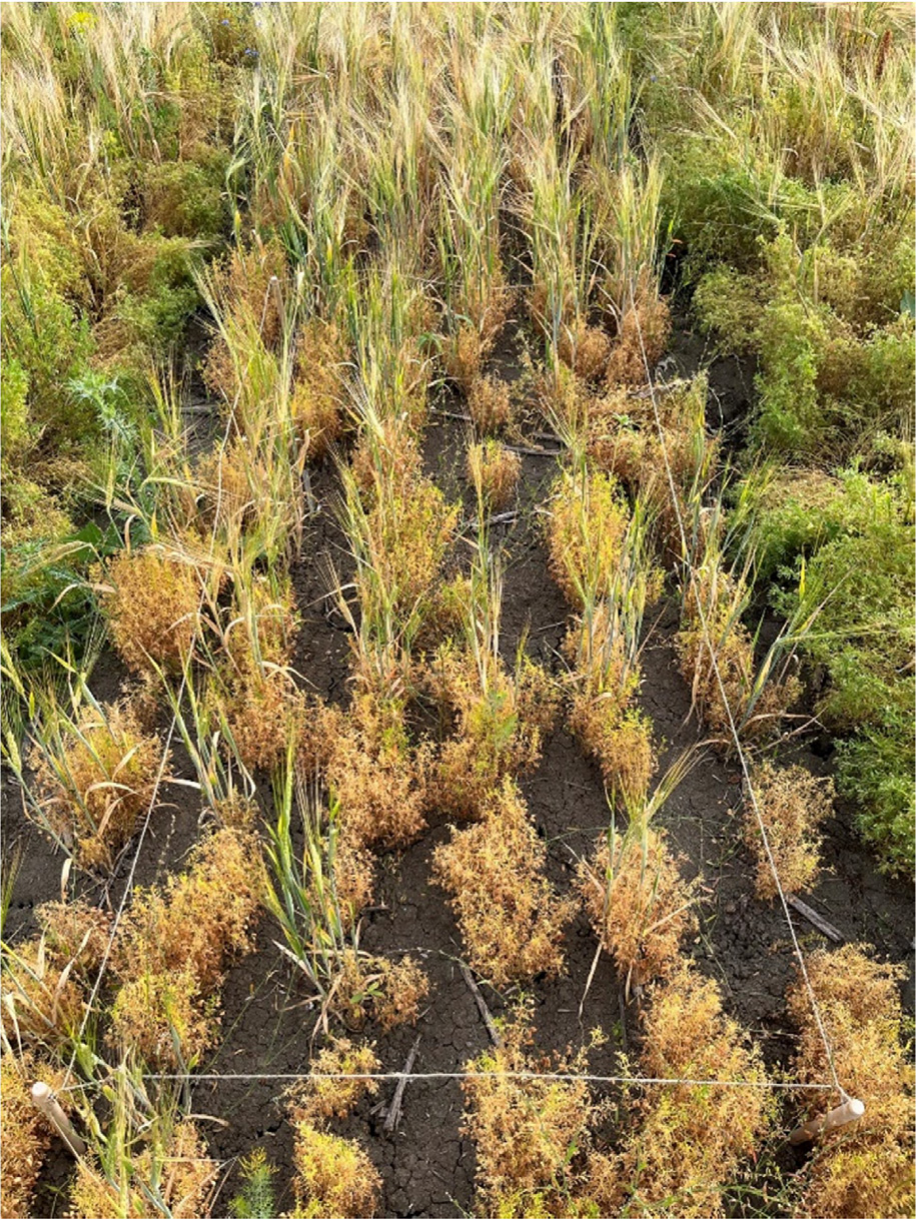
1 × 2 m sample-plots were placed in the middle of the experimental plots (picture: Vollheyde 2022).

As the experimental plots on the field sites differed in size, the wild flora survey was conducted in smaller sample-plots of a standardized size of 1 × 2 m. As shown in Fig. 3, the plots were placed in the middle of each experimental plot, following the drilled lines.

Vegetation surveys were conducted during May-July in 2022 and 2023. Table 3 provides an overview of all parameters that were mapped on-site. Vegetation cover was estimated on an integer scale. If a sample-plot contained small species with extremely low abundance, it was not feasible to accurately estimate true coverage. In such instances, a coverage value of 0.5% was recorded. The nomenclature of flora species was retrospectively standardized based on Euro+Med PlantBase [15].

**Table 3:** Parameters mapped during fieldwork.

Parameter	Explanation
Legume growth stage	BBCH scale, according to [16]
Total plant (crops and wild flora) cover	Freely estimated in %
Cover of each crop	Freely estimated in %
Total cover of all wild flora	Freely estimated in %
Full wild flora species list including individual coverage	Coverage freely estimated in %

The determination of grain yield was done slightly differently in the experimental stations. In all locations, crops were hand-harvested at the crop maturity stage, air dried at room temperature, threshed to separate grain from stalks and husks, and finally, cleaned seeds were weighted. In Spain, the full plots were harvested, whereas in Croatia and Tunisia, grain yield was determined based on samples – 2m² per plot in Croatia, and 10 randomly selected plants per plot in Tunisia. In 2023, the Spanish lentil trials failed due to extreme weather conditions. In the Cuenca, the lack of rainfall during February, March, and April 2023 (total February-April 2023: 21.2 mm, average total of 2013–2022 for February-April: 165.5 mm, [17]) resulted in poor development of the lentils in the months of maximum vegetative growth. In addition, intense rain in the middle of June (2023: 66.4 mm, average total of 2013–2022: 18 mm, [17]), just before the scheduled harvest date, completely devastated the small lentil plants that had survived the intense drought, which had barely developed pods and seeds. Carmona was also affected by an extraordinary drought period with lacks of rainfall during the growing season 2022/2023 (December 2022-April 2023: total rain 32.9 mm, average temperature 15.6 °C [18], December-April 2012–2022: average total rain 193.3 mm, average temperature: 13.6 °C) resulting in poor crop development and no pod formation. Thus, in 2023 in Spain, the crops were not harvested. However, for these trials, only yield and variables related to the legume phase (e.g. weather) are not included in the dataset. Still, management details (except for harvest date), soil data as well as wild flora were still recorded and are presented in the dataset.

In addition to the tested practices (treatments), we synthesized all other associated management practices held constant in the trial, to compile a comprehensive overview of the legume system's management. Thus, the management details encompass information about cultivars and cropping history, sowing density, tillage, fertilization, and management regimes.

To describe environmental factors, we gleaned weather data from local weather stations [17,18]. This comprises total precipitation (in mm) and average temperature (in °C) during the legume phase, starting from the day of legume sowing until the day of harvest. Lastly, sand, silt, and clay content on the experimental sites were determined by laboratory analysis. The exception to this is Cuenca, where the soil texture was analysed using a bottle test [19].

## Limitations

Due to extreme weather conditions in 2023, in some trials the pulses did not form pods, or pods did not mature. In these cases, no production and thus also weather data during the legume phase (due to non-existing harvest date) could be retrieved. Moreover, trials were not standardized across countries and were rather intended to represent potential new management approaches suitable for the respective agronomic and climatic context.

## Ethics Statement

The authors confirm that they have read and followed the ethical requirements for publication in Data in Brief. The authors also confirm that the work does not involve human subjects, animal experiments or any data collected from social media platforms.

## CRediT authorship contribution statement

**Anna-Lena Vollheyde:** Conceptualization, Methodology, Investigation, Data curation, Writing – original draft. **Dulcenombre Rodriguez Navarro:** Conceptualization, Methodology, Investigation, Writing – review & editing, Funding acquisition. **Marcelino de los Mozos Pascual:** Conceptualization, Methodology, Resources, Investigation, Writing – review & editing. **María Cristina Alcántara Ramírez:** Methodology, Investigation, Writing – review & editing. **Sanja Sikora:** Conceptualization, Methodology, Investigation, Writing – review & editing, Funding acquisition. **Ivana Rajnović:** Investigation, Writing – review & editing. **Imran Hammami:** Conceptualization, Methodology, Investigation, Writing – review & editing. **Darine Trabelsi:** Conceptualization, Methodology, Investigation, Writing – review & editing, Funding acquisition. **Christina von Haaren:** Conceptualization, Methodology, Writing – review & editing, Supervision, Funding acquisition.

## Data Availability

Dataset on wild flora diversity and associated yield and agri-environmental information of legume field trials in the Mediterranean (Original data) (Forschungsdaten-Repositorium der Leibniz Universität Hannover)
